# Individual and collective positive health behaviors and academic achievement among U.S. undergraduate students

**DOI:** 10.1371/journal.pone.0323610

**Published:** 2025-06-11

**Authors:** Alyssa M. Lederer, Sara B. Oswalt, Katherine S. Eddens

**Affiliations:** 1 Department of Applied Health Science, Indiana University School of Public Health-Bloomington, Bloomington, Indiana, United States of America; 2 Department of Public Health, University of Texas at San Antonio, San Antonio, Texas, United States of America; 3 Department of Epidemiology and Biostatistics, Indiana University School of Public Health-Bloomington, Bloomington, Indiana, United States of America; King Saud University, SAUDI ARABIA

## Abstract

**Background:**

Behaviors associated with chronic disease can become habituated during young adulthood and may influence students’ academic achievement, affecting their future health and economic prospects. However, more research is needed to understand this relationship. This study therefore examined the connection, both individually and collectively, between undergraduate students’ chronic disease prevention behaviors and academic performance.

**Methods:**

We examined the relationship between 14 positive health behaviors related to diet, physical activity, sedentary screen time, and tobacco product use and cumulative grade point average (GPA; A, B, C, D/F) using the Spring 2023 American College Health Association-National College Health Assessment III (N = 50,792 students; N = 125 institutions). Log binomial regressions produced adjusted prevalence ratios for performing each health behavior based on GPA, controlling for year in school, sex/gender, race/ethnicity, and BMI. A composite health index was also calculated, and multivariate negative binomial regression examined the health index score by GPA category.

**Results:**

Analyses found that A and B GPA categories were significantly different than D/F for all dietary behaviors, all physical activity behaviors, watching TV and gaming, and using vaping products. Students with a higher GPA had significantly more positive health behaviors based on the composite index than each proceeding GPA group.

**Conclusions:**

This study found a relationship between students’ academic achievement and engagement in positive behaviors that prevent or mitigate chronic disease and is the first to examine college students’ health behaviors cumulatively. Initiatives that support college student well-being may benefit students’ academic success as well as reduce chronic disease risk.

## Introduction

The college years are a critical developmental stage in which young adults adopt new health habits, establishing key behavioral patterns that influence future chronic disease risk [1,2]. At the elementary and secondary levels, substantial research has found that in addition to health being important in its own right, students’ health is also linked to their academic performance. This has led several reviews to conclude that schools are a key setting for health promotion efforts, and targeting students’ well-being and academic success should yield mutually beneficial outcomes [3,4]. Institutions of higher education are likewise a crucial environment for public health initiatives given institutions’ imperative to support student success and the ability to leverage existing resources [1,5,6]. Even more, colleges and universities are considered their own communities and have the capability to reach the almost 19 million students enrolled annually [7], including almost 40% of the 18–24-year-old U.S. population [8].

Understanding factors that contribute to college students’ educational attainment is essential. College graduation is considered a social determinant of health, positively impacting people’s future health, employment, and economic outcomes [9]; yet U.S. graduation rates are suboptimal [10]. Unlike their younger counterparts, little information synthesizing the relationship between college students’ health and academic performance has been available until recently. A newly published comprehensive scoping review found preliminary evidence indicating a relationship between students’ health and academic achievement. Yet the review also determined that more rigorous research, particularly with a national scope, is needed [11].

To fill the gap for more robust postsecondary research, we previously replicated a study conducted by Rasberry and colleagues [12] investigating the connection between high school students’ health behaviors and grades using the Youth Risk Behavior Survey (YRBS). Modeling their methods, we examined the link between college students’ cumulative grade point average (GPA) and 30 health behaviors using a national college health dataset. Similar to Rasberry et al.’s original research [12], we found that college students’ protective health behaviors were associated with higher GPAs whereas their risk-related behaviors were mostly associated with lower GPAs [13].

As a follow-up to the Rasberry et al. study [12], the same research group, now led by Hawkins, published another study using more recent YRBS data to assess the relationship between high school students’ positive health behaviors that alleviate chronic disease risk and their academic performance. Hawkins et al. described the need to examine this relationship both independently and collectively, noting no other studies had examined young people’s health behaviors *cumulatively* in connection to grades [14]. This study yielded similar results to their prior research, but with the new finding that cumulative health protective behaviors were also associated with better academic performance among high school students. Given the same gap exists in the U.S. collegiate literature [13], we now replicate this successive study by Hawkins and colleagues but focus on undergraduate students to examine, individually and collectively, the same positive health behaviors that can reduce chronic diseases, the leading causes of morbidity and mortality in the U.S. [15].

Our study had two primary objectives: 1) To update the understanding of relationships between *individual* health-promoting behaviors connected to chronic disease risk (dietary, physical activity, sedentary screen time, and tobacco product use behaviors) and cumulative GPA among undergraduate students; and 2) To *collectively* examine the relationship between these health-promoting behaviors and undergraduate students’ cumulative GPA. In doing so, we aim to provide up-to-date and novel information that can inform efforts targeting undergraduate students’ well-being and academic success.

## Materials and methods

### Design and sample

The current study is based on Hawkins and colleagues’ methods [14] and uses data from the American College Health Association-National College Health Assessment III (ACHA-NCHA III). The ACHA-NCHA III is a cross-sectional comprehensive online college health survey administered by the American College Health Association (ACHA) in cooperation with self-selected institutions of higher education. More information about the ACHA-NCHA III can be found elsewhere [16]. Administered during the fall and spring annually, institutions receive their campus-specific results and all schools that conduct a census or random sample are aggregated into a national dataset grouped by the fall or spring survey period, available for secondary analysis by request to ACHA.

We used the Spring 2023 national ACHA-NCHA III dataset. There was an 11% response rate, consistent with other college health surveys of similar scope [17,18]. We restricted the sample to full-time undergraduates who reported a GPA. The final analytic sample was N = 50,792 students at 125 institutions. Schools must receive institutional review board (IRB) approval from their own institution prior to participation and students provide informed consent before participating in accordance with their institution’s policies. All students must be age 18 or older to participate. The present study used anonymized national secondary data made available by ACHA and was deemed not human subjects research by Indiana University’s IRB due to it being deidentified secondary analysis.

### Measures

All variables used in the current study and the operationalization of response options paralleled those in Hawkins et al.’s original research [14] to the degree possible. For health behaviors associated with chronic disease, there were three dietary behaviors, three physical activity behaviors, three sedentary screen time behaviors, and five tobacco product use behaviors (14 behaviors in total). For dietary behaviors, students were asked about their fruit, vegetable, and sugar-sweetened beverage consumption. For physical activity behaviors, students reported their aerobic physical activity and muscle strengthening activity to ascertain if they met U.S. recommended guidelines [19] and if they participated in organized college athletics. For sedentary behaviors, students were asked about screen time use, explicitly time spent watching TV, gaming, and using social media. For tobacco product use behaviors, students reported their use of cigarettes, e-cigarettes or other vape products, chewing or smokeless tobacco, cigars or little cigars, and water pipe or hookah. Table 1 provides the questions and analytic coding for all health behaviors. Measures were assessed for content validity and underwent multiple phases of review during the ACHA-NCHA III development process [16], and ACHA-NCHA III screen time and tobacco-related questions were taken from existing sources [20,21].

**Table 1:** Questions and analytic coding for positive health behaviors, ACHA-NCHA III, 2023.

Variable	Question^a^	Analytic Coding
***Dietary Behaviors***		
Ate fruit or drank 100% fruit juices one or more times per day	In the last 7 days, how many servings of fruit did you eat on average per day? One serving is a medium piece of fresh fruit, ½ cup of fresh, frozen, or canned fruit; ¼ cup of dried fruit; or ¾ cup of 100% fresh fruit juice	Ate fruit or drank 100% fruit juice ≥1 times per day vs. did not eat fruit or drink 100% fruit juice
Ate vegetables one or more times per day	In the last 7 days, how many servings of vegetables did you eat on average per day? One serving is ½ cup of fresh, frozen, or canned vegetables; ¾ cup 100% vegetable juice; or 1 cup salad greens	Ate vegetables ≥1 times per day vs. did not eat vegetables
Did not drink a sugar-sweetened beverage	In the last 7 days, how many servings of sugar-sweetened beverages did you drink on average per day? One serving is 12 oz of soda; 8 oz of sugar-sweetened, flavored water or sports drink; 6 oz of sugar-sweetened coffee, tea, or juice.	Did not drink sugar-sweetened beverages vs. did drink sugar-sweetened beverages ≥1 times per day
***Physical Activity Behaviors***		
If met US recommended guideline for aerobic activity for adults^b^	Based on two questions: For the next two questions, the levels of physical activity intensity can be characterized in terms of breathing difficulty. A person doing moderate physical activity can typically talk, but not sing while doing the activity. A person doing vigorous physical activity typically cannot say more than a few words without pausing for a breath while doing the activity. In the last 7 days, how many (total) minutes did you spend doing moderate physical activity? Examples: brisk walking, dancing, or household chores. In the last 7 days, how many (total) minutes did you spend doing vigorous physical activity? Examples: running, swimming laps, or hiking.	Used both items to calculate if met US recommended guideline for aerobic physical activity and coded as met US recommended guideline for aerobic physical activity vs. did not meet US recommended guideline
If met US guideline for muscle strengthening activity^c^	In the last 7 days, on how many days did you do exercises to strengthen or tone your muscles? Examples: push ups, sit ups, or weightlifting/training.	Met US recommended guideline for muscle strengthening vs. did not meet US recommended guideline for muscle strengthening activity
Played on at least one sports team	Do you participate in organized college athletics at any of the following levels? Varsity, Club sports, Intramurals	Played at ≥1 athletic levels vs. none
***Sedentary Behaviors***		
Did not watch television for more than 15 hours per week^d^^,^^e^	How many hours do you spend in a typical week (7 days) on the following activities? Watching TV, streaming movies/TV or other media or entertainment	≤ 15 hours vs. more than 15 hours per week
Did not game for more than 15 hours per week^d^^,^^e^	How many hours do you spend in a typical week (7 days) on the following activities? Gaming	≤ 15 hours vs. more than 15 hours per week
Did not use social media for more than 15 hours a week^d^^,^^e^	How many hours do you spend in a typical week (7 days) on the following activities? Using social media	≤ 15 hours vs. more than 15 hours per week
***Tobacco Product Use Behaviors***		
Did not smoke cigarettes^f^	Based on 2 questions: In your life, which of the following substances have you ever used? Tobacco or nicotine delivery products (cigarettes, ecigarettes, Juul or other vape products, water pipe or hookah, chewing tobacco, cigars, etc.) If answered yes to the above screening question: Within the last 3 months, which tobacco products have you used? Cigarettes	Used both items to determine if used product in last 3 months (i.e., combined never used with did not use in last 3 months) and coded as did not use cigarettes vs. used cigarettes in the last 3 months
Did not use electronic vapor products^f^	Based on 2 questions: In your life, which of the following substances have you ever used? Tobacco or nicotine delivery products (cigarettes, ecigarettes, Juul or other vape products, water pipe or hookah, chewing tobacco, cigars, etc.) If answered yes to the above screening question: Within the last 3 months, which tobacco products have you used? E-cigarettes or other vape products (for example: Juul, etc.)	Used both items to determine if used product in last 3 months (i.e., combined never used with did not use in last 3 months) and coded as did not use electronic vapor products vs. used electronic vapor products in the last 3 months
Did not use smokeless tobacco^f^	Based on 2 questions: In your life, which of the following substances have you ever used? Tobacco or nicotine delivery products (cigarettes, ecigarettes, Juul or other vape products, water pipe or hookah, chewing tobacco, cigars, etc.) If answered yes to the above screening question: Within the last 3 months, which tobacco products have you used? Chewing or smokeless tobacco in last 3 months	Used both items to determine if used product in last 3 months (i.e., combined never used with did not use in last 3 months) and coded as did not use smokeless tobacco vs. used smokeless tobacco in the last 3 months
Did not use cigars, cigarillos, or little cigars^f^	Based on 2 questions: In your life, which of the following substances have you ever used? Tobacco or nicotine delivery products (cigarettes, ecigarettes, Juul or other vape products, water pipe or hookah, chewing tobacco, cigars, etc.) If answered yes to the above screening question: Within the last 3 months, which tobacco products have you used? Cigars or little cigars	Used both items to determine if used product in last 3 months (i.e., combined never used with did not use in last 3 months) and coded as did not use cigars, cigarillos, or little cigars vs. used cigars, cigarillos, or little cigars in the last 3 months
Did not use water pipe or hookah^f^	Based on 2 questions: In your life, which of the following substances have you ever used? Tobacco or nicotine delivery products (cigarettes, ecigarettes, Juul or other vape products, water pipe or hookah, chewing tobacco, cigars, etc.) If answered yes to the above screening question: Within the last 3 months, which tobacco products have you used? Water pipe or hookah	Used both items to determine if used product in last 3 months (i.e., combined never used with did not use in last 3 months) and coded as did not use water pipe or hookah vs. used water pipe or hookah in the last 3 months

^a^ All questions and complete response options can be found elsewhere [22].

^b^ U.S. recommended guideline for physical activity for aerobic activity is 150 + minutes per week of moderate aerobic activity, where 1 minute of vigorous activity equals two minutes of moderate activity [19].

^c^ U.S. recommended guideline for muscle strengthening activity is at least two days of muscle strengthening [19].

^d^ These ACHA-NCHA III questions were adapted from the Student Experience in the Research University (SERU) survey [21].

^e^ Hawkins et al. [14] original response options were ≥ 2 hours per day vs. more than 2 hours per day based on the American Academy of Pediatrics recommendation for screen time with the use of media for adolescents, which is no longer intact. New recommendations do not include a set screen time limit [23]. We matched the prior response options using hours spent in a typical per week based on categories available in the ACHA-NCHA III.

^f^Questions were taken from the ASSIST [20], a question set embedded in the ACHA-NCHA III.

All behaviors were comparable to the original study [14], with the following exceptions given differing instrumentation. Regarding diet, the ACHA-NCHA III did not include breakfast, milk, and water consumption, and it combined soda and sports drinks (whereas the YRBS measured each separately). For physical activity, attending physical education classes was not included and the U.S. recommended guideline for muscle strengthening was added. For screen time behaviors, gaming and social media use were measured separately (versus combined in the YRBS), and hours were reported by week rather than day. All tobacco use product behaviors were the same, with water pipe or hookah added, although measured as last three months rather than last 30 days. Ultimately, our study included an index for 14 behaviors whereas the original study examined 16. The cumulative health behavior index was created by dichotomizing each individual positive health behavior as 0 (did not perform) versus 1 (did perform), for a total score range of 0–14.

Students’ self-reported cumulative GPA was measured by the question, “What is your approximate cumulative grade average?” with possible responses: A+, A, A−, B+, B, B−, C+, C, C−, D+, D, D−, F, N/A. Responses were consolidated into single letter GPAs (A, B, C, combined D/F) as per the grade categories employed by Hawkins et al. [14]. Self-reported sociodemographic covariates were undergraduate year in school; a sex/gender variable computed by ACHA [22]; race/ethnicity, in which students could check all responses that applied (students who selected multiple identities were re-coded into the multiracial category); and body mass index (BMI) category. All sociodemographic covariate response categories used in the present study can be found in Table 2. ACHA calculated estimated BMI from students’ self-reported height and weight and then recoded it into the following categories per Centers for Disease Control and Prevention (CDC) guidelines [24]: < 18.5 underweight, 18.5–24.9 healthy weight, 25–29.9 overweight, 30–34.5 class 1 obesity, 35–39.9 class II obesity, ≥ 40 Class III obesity; all obesity classes were consolidated into a single obesity group. As per the original study, BMI was included given evidence that it has a weak but negative association with academic performance among high school and postsecondary students [25]. Complete questions and original response options for all measures used in the current study can be found in the ACHA-NCHA III instrument [22].

**Table 2:** Participant characteristics, ACHA-NCHA III Spring 2023 (N = 50,792).

Characteristic	n (%)
*Cumulative Grade Point Average*	
A	30473 (60.0)
B	16789 (33.1)
C	3227 (6.4)
D/F	303 (0.6)
*Year in School*	
1st year undergraduate	14211 (28.0)
2nd year undergraduate	12117 (23.9)
3rd year undergraduate	12953 (25.5)
4th year undergraduate	9673 (19.0)
5th or more year undergraduate	1838 (3.6)
*Sex/Gender (n = 50327)*	
Cisgender Female	33554 (66.7)
Cisgender Male	13494 (26.8)
Transgender/Gender non-conforming	3279 (6.5)
*Race/Ethnicity (n = 50574)*	
White	27984 (55.3)
Black or African American	2142 (4.2)
Asian or Asian American	7272 (14.4)
Latino/a/x	5147 (10.2)
All other races/ethnicities and multi-racial	8029 (15.9)
*Body Mass Index (BMI) Category (n = 49585)*	
Healthy weight	28529 (57.5)
Underweight	2880 (5.8)
Overweight	10782 (21.7)
Obese	7394 (14.9)

### Analysis

Analyses replicated those conducted by Hawkins, et al. [14] as closely as possible, with some deviations explicated. Data analyses were generated using SAS software version 9.4. We initially conducted multivariable logistic regression analysis with each health behavior with GPA as the primary dependent variable (using D/F GPA category as the referent) and adjusting for sex/gender, race/ethnicity, year in school, and BMI. As logistic regression often overestimates prevalence odds ratios of common outcomes (prevalence greater than 10%) and the health behaviors in this sample were all common (most with a prevalence >70%), we also conducted log-binomial regressions to estimate prevalence ratios directly, using the GENMOD command with binomial distribution and log link function [26–30]. Each student record in the sample contained an anonymized school ID; therefore we clustered standard errors of participants by their school ID to account for the possibility of any unobservable effects influencing variance within the same institution [30].

The log-binomial models the probability of the outcome for individual *i* in cluster *j* (p_ij) as


log(p_ij) = β0 + β1 * X_ij +  γ * Cluster_j + ... + other covariates

where β0 is the intercept, *β1* is the coefficient for the exposure variable *X_ij,* γ is the coefficient for the cluster effect, and *Cluster_j* is the indicator variable for cluster j. Then, the estimated Prevalence Ratio (PR) is represented by the exponentiated coefficient of *β1*, the exposure variable, and indicates the change in prevalence of the outcome associated with a one unit increase in the exposure, adjusted for other covariates and clustering. Applied to these data, we model the change in prevalence of each healthy behavior associated with the unit of GPA in comparison to a D/F GPA, adjusting for year in school, race/ethnicity, sex/gender, BMI category, and clustering by institution, where:


$$\begin{array}{cc} log(p\_ij)=\;\beta 0\;+\;\beta 1\;*\;GPA_{ij}+\;\gamma \;*\;Cluster_{institutionID}+\beta 2\;*\;Year\;in\;school\\ & +\;\beta 3\;*race/ethnicity+\;\beta 4\;*\;Sex/gender+\;\beta 5\;*\;BMI\;category \end{array}$$

This produced more conservative adjusted prevalence ratio estimates using an appropriate method for the field of epidemiology; therefore, we report these results rather than the multivariable logistic regression results. Total prevalence counts and proportions for each positive health behavior are reported as well as adjusted prevalence ratios (aPRs), 95% confidence intervals (95% CIs), and p-values.

A composite health index score was created to represent the total count of positive health behaviors that participants engaged in. Each positive health behavior (n = 14) was designated 0 = the participant *did not* perform the behavior or 1 = the participant *did* perform the behavior. Participant behaviors were then summed to create a composite health index score ranging between 0 and 14. Hawkins et al. [14] conducted a factor analysis and confirmed all individual health behaviors they examined were appropriate for inclusion in the composite health index. Because we selected the ACHA-NCHA III items that best represented the YRBS measures used by Hawkins, et al. [14] to best replicate the original study, we chose to include all 14 identified equivalent behaviors in our composite index rather than conduct a factor analysis to identify which behaviors to include and risk losing some comparison measures.

Modeling the work of Hawkins et al. [14], we examined the relationship of GPA and cumulative preventative health behaviors with the composite health index score serving as the dependent variable. We used multivariate negative binomial regression which is appropriate for modeling over-dispersed count outcome variables. The log of the count outcome is predicted with a linear combination of the predictors, allowing for the interpretation of coefficients as multiplicative effects. The negative binomial model includes a dispersion parameter to account for overdispersion in its estimation of the count. The linear equation is similar to the log-binomial:


$$\log\left(\mu_{i}\right)=\;\beta 0\;+\;\beta 1*X_{i}1\;+\;...\;+\;\beta pXip\;+\;\gamma \_j$$

where $\mu_{i}$ is the expected count for observation *i*, β0 is the intercept, *β1…*βp are the coefficients for the predictor variables $X_{i}1$*…*Xip, and γ_j is the cluster-specific random effect to produce standard errors that are robust to clustering within groups. For our model, we specified the negative binomial regression with log link function for count data and standard errors clustered by institution ID, which calculates the predicted count of health behaviors at each level of our sociodemographic variables and GPA, holding all other variables in the model at their means. For example: $\;\mathrm{-2pc}\backslash (\log\left(\mu_{i}\right)=\;Intercept\;+\;\beta 1*GPA\;+\;\beta 2*Year\;in\;school+\;\beta 3\;*Race/ethnicity+\;\beta 4\;*\;Sex/gender+\;\beta 5\;*\;BMI\mathrm{\backslash break}\;3pccategory+\;\gamma \_institutionID$

Exponentiating $\mu_{i}$ then provides the predicted count of health behaviors in the composite score for each level of the predictor variable.

The margins macro in SAS fits the generalized linear or GEE model specified and estimates the average marginal effects for variables in the model. Using the *%margins* command, we generated multivariable negative binomial regression models with the log link function for count data and robust standard errors to report the *differences* in the composite score of positive health behaviors for each sociodemographic variable and GPA, adjusting for all other variables in the model. Average marginal effects (AME) and standard errors (SE) are reported as well as the p-value for differences in marginal effects. We included all cases with complete data for all variables in each specific model. The percentage of missing cases in the individual log binomial models for health behavior prevalence ranged from 3.5% to 4.6%, all within an acceptable rate of missing less than 5%. We document count and proportions of health behavior engagement (yes/no) for each model and provide the total n included in each log binomial model. Of a total of 48,484 participants with complete data for the health composite score, 47,011 of those had complete data for all variables included in the negative binomial model. This is, therefore, the total sample for the negative binomial models, representing 97% of possible cases with a health composite score.

## Results

### Participant characteristics

Descriptive statistics for student participants are reported in Table 2. Respondents mostly reported an average cumulative GPA of A (60.0%) or B (33.1%) and were distributed similarly across undergraduate years one through four. Two-thirds of respondents were cisgender female (66.7%), and a little over half self-identified only as White (55.3%) and had a healthy BMI (57.5%).

### Individual positive health behaviors

The prevalence of each positive health behavior is reported in Table 3, with engaging in the behavior coded as “Yes” and not engaging in the behavior as “No.” Adjusted prevalence ratios for each positive health behavior by reported GPA are also included in Table 3, with GPA of “D/F” as the referent, adjusted for year in school, race/ethnicity, sex/gender, BMI category, and clustered by institution.

**Table 3:** Prevalence of participants reporting each positive health behavior and adjusted prevalence ratios (aPRs) from log-binomial regression, by cumulative grade point average (D/F as referent), ACHA-NCHA III Spring 2023.

Healthy Behavior	Engaged in healthy behavior	aPR (95% CI)†		
	Yes, n (%)	p-value		
	No, n (%)	A	B	C
*Dietary behaviors*				
Ate fruit one or more times per day, on average	41016 (83.8) 7944 (16.2) n = 48,960	1.14 (1.06, 1.22) **.0002**	1.11 (1.04, 1.19) **.002**	1.06 (0.99, 1.14) .082
Ate vegetables one or more times per day, on average	43258 (88.3) 5725 (11.7) n = 48,983	1.11 (1.04, 1.17) **.0006**	1.08 (1.02, 1.14) **.008**	1.03 (0.97, 1.08) .391
Drank no sugar-sweetened beverages on average per day	13073 (26.8) 35665 (73.2) n = 48,738	1.78 (1.30, 2.45) **.0003**	1.45 (1.05, 1.99) **.023**	1.23 (0.88, 1.72) .221
*Physical activity behaviors*				
Met US recommended guidelines for aerobic physical activity (150 + min/week)	34695 (71.5) 13830 (28.5) n = 48,525	1.18 (1.06, 1.30) **.001**	1.14 (1.03, 1.26) **.009**	1.08 (0.98, 1.19) .115
Met US recommended guidelines for muscle strengthening (2 + days/week)	24134 (49.2) 24873 (50.8) n = 49,007	1.23 (1.08, 1.41) **.002**	1.24 (1.08, 1.42) **.002**	1.14 (0.99, 1.31) .065
Participated in college athletics at varsity, club sports, or intramural levels	10143 (20.9) 38297 (79.1) n = 48,440	1.56 (1.18, 2.07) **.002**	1.50 (1.13, 1.99) .**005**	1.26 (0.94, 1.68) .125
*Sedentary behaviors*				
Did not watch television for more than an average of 2 hours a day	43414 (88.7) 5525 (11.3) n = 48,939	1.16 (1.09, 1.24) **<.0001**	1.14 (1.07, 1.21) **<.0001**	1.10 (1.03, 1.16) **.003**
Did not play video games for more than an average of 2 hours a day	46893 (95.9) 2008 (4.1) n = 48,901	1.06 (1.03, 1.09) **.0002**	1.05 (1.02, 1.09) **.001**	1.04 (1.01, 1.07) **.020**
Did not use social media for more than an average of 2 hours a day	42343 (87.0) 6346 (13.0) n = 48,689	1.05 (1.00, 1.10) .072	1.02 (0.97, 1.07) .418	0.99 (0.94, 1.04) .750
*Tobacco product use in the past 3 months*				
Did not use cigarettes	44291 (90.2) 4788 (9.8) n = 49,079	1.05 (1.00, 1.10) .055	1.03 (0.99, 1.08) .185	1.00 (0.96, 1.05) .968
Did not use e-cigarettes or other vape products	39922 (81.3) 9157 (18.7) n = 49,079	1.21 (1.13, 1.30) **<.0001**	1.13 (1.05, 1.21) **.0008**	1.05 (0.98, 1.13) .175
Did not use chewing or smokeless tobacco	48255 (98.3) 824 (1.7) n = 49,079	1.01 (0.99, 1.02) .317	1.00 (0.99, 1.02) .744	1.01 (0.99, 1.02) .317
Did not use cigars or little cigars	47878 (97.6) 1201 (2.4) n = 49,079	1.01 (0.99, 1.02) .398	1.00 (0.98, 1.02) .727	0.99 (0.97, 1.01) .433
Did not use water pipe or hookah	48160 (98.1) 919 (1.9) n = 49,079	1.02 (1.00, 1.03) .070	1.01 (0.99, 1.02) .470	1.00 (0.98, 1.02) .827

†adjusted Prevalence Ratio and 95% Confidence Interval. Log-binomial regression clustered by institution and adjusted for year in school, sex/gender, race/ethnicity, and BMI category.

Students who reported an A GPA were more likely to engage in healthy dietary behaviors in comparison to students reporting a D/F GPA (all p < .001). Specifically, compared to students reporting a D/F GPA, students reporting an A GPA were 14% more likely to eat fruit every day, 11% more likely to eat vegetables daily, and 78% more likely to *not* drink a sugar sweetened beverage daily. Students reporting a B GPA were also more likely to engage in healthy dietary behaviors than students reporting a D/F GPA, but there was no significant difference in prevalence of dietary behaviors between students reporting C and D/F GPAs.

The prevalence of healthy physical activity behaviors was also significantly higher among students reporting A and B GPAs compared to students reporting D/F GPAs, but there were no significant differences between students with C and D/F GPAs. Students with an A GPA were 18% more likely to meet the U.S. recommended guidelines for aerobic physical activity (p = .001), 23% more likely to meet U.S. recommended guidelines for muscle strengthening (p = .002), and 56% more likely to participate in college athletics (p = .002) than those with D/F GPAs. Similarly, healthy physical activity behaviors were higher among students reporting a B GPA compared to those reporting D/F GPAs (aerobic activity 14% higher, p = .009; muscle strengthening 24% higher, p = .002; college athletics participation 50% higher, p = .005). Again, there was no significant difference in prevalence of physical activity behaviors between students with C and D/F GPAs.

For sedentary behaviors, students with A, B, and C GPAs were statistically significantly more likely to watch television and play video games <15 hours a week than students reporting D/F GPAs. Compared to students with a D/F GPA, students reporting an A GPA were 16% more likely to watch ≤15 hours of television per week (p < .0001), students with Bs were 14% more likely (p < .0001), and students with Cs were 10% more likely (p = .003) to watch <15 hours of television per week. Students with A GPAs were 6% more likely, those with B GPAs were 5% more likely, and those with C GPAs were 4% more likely than those with D/F GPAs to play video games for ≤15 hours a week (p = .0002, p = .001, p = .020, respectively). There were no statistically significant differences between GPA categories in using social media for ≤15 hours a week.

Among tobacco use behaviors, only one behavior – did not use e-cigarettes or other vape products in the past 3 months – had a prevalence less than 90% among the total sample; this was the only tobacco use behavior with statistically significant differences in prevalence among GPA groups. Compared to students reporting a D/F GPA, there was a 21% higher prevalence of no reported e-cigarette or vape use in the past three months among students reporting an A GPA (p < .0001) and a 13% higher prevalence among students reporting a B GPA (p = .0008). All results are displayed in Table 3.

### Collective positive health behaviors

**Fig 1:**
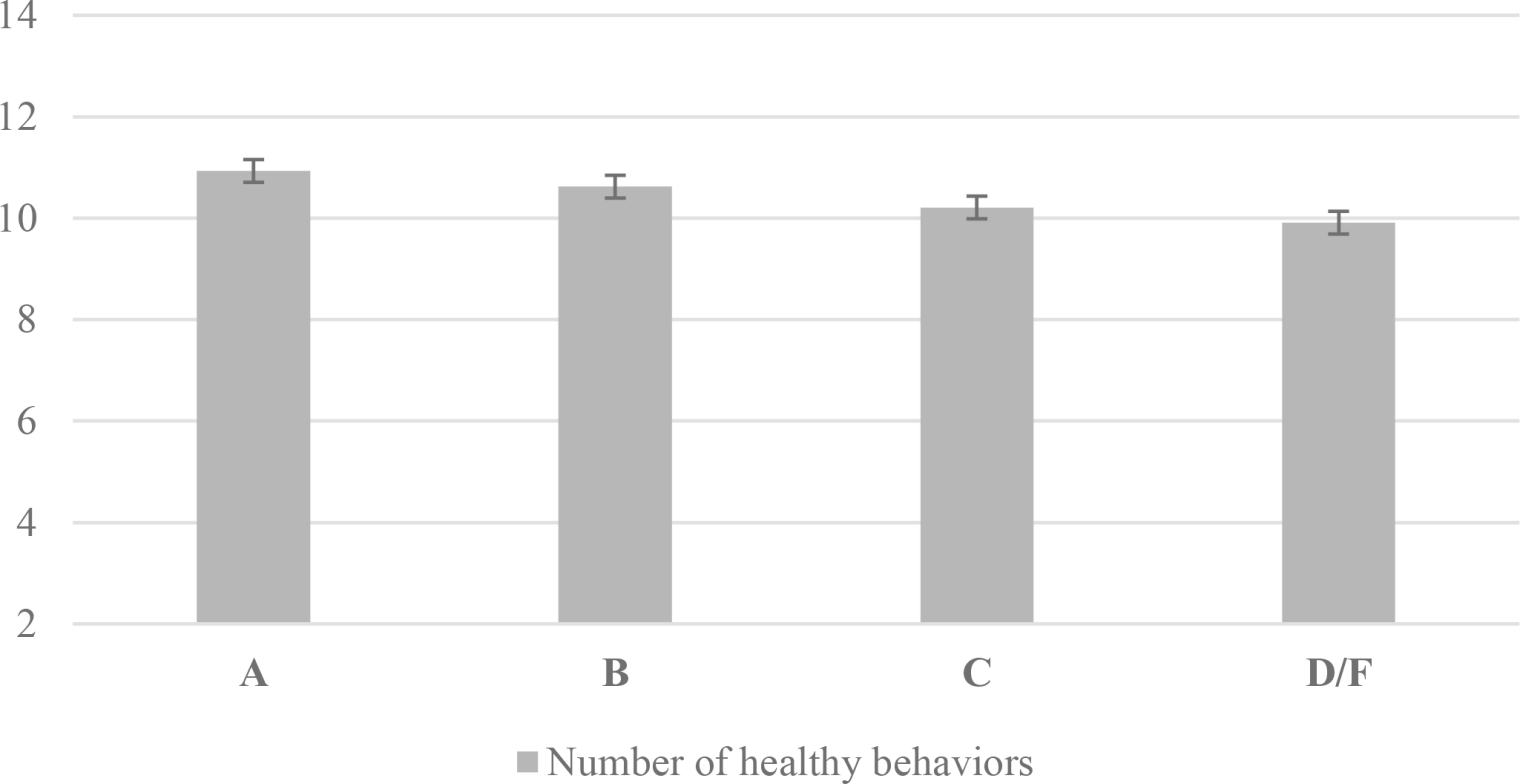
Participants’ unadjusted number of healthy behaviors based on the composite health index by GPA level (N = 48,484).

The composite health index score ranged from 2 to 14, with a mean of 10.78. As shown in Fig 1, the unadjusted mean composite score was significantly different between every level of GPA (Welch’s ANOVA F(3,5207)=521.17, p < .0001):10.93 (95% CI: 10.91, 10.96) for students reporting an A, 10.62 (95% CI: 10.60, 10.65) for B, 10.21 (95% CI: 10.16, 10.25) for C, and 9.91 (95% CI: 9.78, 10.03) for D/F.

The average marginal effects and standard errors of each level of sociodemographic variables generated by the multivariable negative binomial regression are displayed in Table 4. With adjustment for all other covariates and with standard errors clustered by institution, students who reported an A GPA had, on average, 1.02 (p < .0001) more positive health behaviors than students reporting a D/F GPA, students with a B GPA had 0.72 more positive health behaviors (p < .0001), and students reporting a C GPA had an average of 0.32 more healthy behaviors (p = .005) than students reporting a D/F GPA. Students in the first four years of their undergraduate program had a fraction more positive health behaviors than 5^th^ year+ undergraduates, with a decreasing trend from 1^st^ to 4^th^ years (all p < .001, see Table 4). There were significant differences among sex/gender groups with cisgender males having 0.18 more and transgender/gender nonconforming students having 0.52 fewer healthy behaviors than cisgender females (p < .0001 for both). Students in underweight, overweight, and obese BMI categories had significantly fewer positive heath behaviors than those in the healthy weight BMI category (-.50, p < .001; -.07, p = .002; -.54, p < .0001, respectively). Black or African American identifying students had 0.15 fewer healthy behaviors than White identifying students (p < .001), but there were no significant differences between White and other race/ethnicity categories.

**Table 4:** Multivariable negative binomial regression for the number of healthy behaviors among participants, ACHA-NCHA III Spring 2023, N = 47,011.

Characteristic	AME (SE)*	p-value, margins differences
*Cumulative Grade Point Average*		
A	1.02 (.11)	**<.0001**
B	.72 (.11)	**<.0001**
C	.32 (.12)	**.005**
D/F	REF	
*Year in School*		
1st year undergraduate	.24 (.05)	**<.0001**
2nd year undergraduate	.22 (.05)	**<.0001**
3rd Year undergraduate	.18 (.04)	**<.0001**
4th year undergraduate	.15 (.05)	**<.001**
5th or more year undergraduate	REF	
*Sex/Gender*		
Cisgender female	REF	
Cisgender male	.18 (.03)	**<.0001**
Transgender/Gender non-conforming	−.52 (.03)	**<.0001**
*Race/Ethnicity*		
White	REF	
Black or African American	−.15 (.05)	**<.001**
Asian or Asian American	.03 (.04)	.408
Latino/a/x	.003 (.05)	.955
All other race/ethnicities and multi-racial	.01 (.03)	.795
*Body Mass Index (BMI) Category*		
Healthy weight	REF	
Underweight	−.50 (.03)	**<.0001**
Overweight	−.07 (.02)	**.002**
Obese	−.54 (.03)	**<.0001**

* Multivariable negative binomial regression adjusted for GPA, year in school, sex/gender, race/ethnicity, and BMI category, standard errors clustered by institution.

## Discussion

This study examined the relationship between undergraduate college students’ positive health behaviors associated with chronic disease prevention and academic achievement specifically modeling Hawkins and colleagues’ [14] work focused on high school students. In addition to assessing the connection between 14 individual health behaviors and GPA, our research included a unique composite health index to assess students’ comprehensive chronic disease protection status.

Regarding individual health behaviors, the results demonstrate a relationship between positive health behaviors and academic outcomes when comparing students with an A or B GPA to those with a D/F for most behaviors – specifically healthy eating, optimal physical activity, and time spent using TV and video games. Significant findings linking grades and positive health behaviors were less common for students with a C GPA. Additionally, the connection between GPA and tobacco use was less consistent. Refraining from e-cigarettes/vaping was the only tobacco behavior connected to GPA, with students with an A or B GPA being significantly more likely not to smoke e-cigarettes/vape than students with a D/F GPA. The connection between e-cigarettes/vaping is particularly pertinent given that while cigarette use is reportedly going down, e-cigarette use is increasing with the highest prevalence rate among individuals ages 18–24 [31].

The significant differences between students with an A and B GPA, but not C GPA, in comparison to a D/F GPA warrants further consideration, particularly in the collegiate context. One explanation may be that a cumulative C GPA is required to avoid academic probation or dismissal and for graduation at many institutions. With the low number of students reporting a D/F GPA on the ACHA-NCHA III, it is possible that students with lower GPAs may have been less likely to respond to the survey, or students who would otherwise have a D/F GPA may have already left college and those remaining may more closely resemble students in the C GPA group in terms of both academic performance (GPA falling below 2.0 once or twice) and health behaviors. This provides a unique opportunity for the development of both academic and health interventions focusing on students with C, D, and F GPAs which could mutually impact both aspects of these students’ lives. Further research should be conducted with students who have left college to understand more specifically how health may have affected their academics.

Overall, this study’s findings are similar to the results of Hawkins et al. [14]. However, direct comparisons should be made with caution given the different instrumentation and item phrasing despite similar concepts measured. Further, the studies focus on two student populations with distinct developmental differences [2]. In comparison to other studies done specifically with college students, many of the current study’s results are similar to examinations of individual health behaviors and academic outcomes using national ACHA-NCHA data collected prior to the COVID-19 pandemic. These studies found that healthy dietary behavior [13,32,33], sufficient physical activity [13,33], refraining from tobacco use [13,34] and limited sedentary behavior [13] were all connected to college students’ GPA. However, studies conducted at single institutions have yielded mixed results regarding the connection between college students’ prevention behaviors and academic performance. Whereas some have found that more physical activity [35] and strength training [36], and refraining from cigar use [37] were associated with better grades, other studies did not find this connection for physical activity [38–40], sedentary behavior [38], or tobacco use [41].

While there has been substantial concern about the toll of pervasive social media use among young people [42], the connection between social media and academic success is less clear with both negative and null results common, prompting recommendations for additional research [43]. It is therefore unsurprising that the current study did not find an association between hours of social media use and GPA. One reason may be that we measured social media as a proxy for sedentary activity, whereas other studies used different measures of social media use [43,44] or academic outcomes [43], or considered mediating factors [45,46].

A unique aspect of the current study, following Hawkins and colleagues’ [14] example, is the composite health index created to examine the relationship between cumulative chronic disease prevention behaviors and academic achievement. The composite results, for both the unadjusted model and the model controlling for student sociodemographics, found that individuals earning an A, B, or C GPA had a significantly higher composite health index score compared to individuals with a cumulative D/F GPA, with more collective positive health behaviors associated with each increasing GPA category. The nuances identified in the adjusted model reveal that intersectionality of student sociodemographics and health behaviors may be crucial to truly understand risk and impact on academics. For example, students’ overall engagement in chronic disease prevention behaviors was lower for those beyond their first year and for male and transgender/gender non-conforming students. There may be a relationship between student sociodemographic characteristics and health behaviors or between student sociodemographic characteristics and GPA that is more prominent than the direct relationship between health behaviors and GPA.

This study has multiple strengths – national scope, the first to examine the connection between *cumulative* chronic health prevention behaviors and academics among college students, and utilization of a rigorous methodology. At the same time, there are some limitations. While the ACHA-NCHA III is one of the largest and most comprehensive sources that assesses college students’ health behaviors nationally, it is not representative of all U.S. college students or institutions of higher education. For instance, participants who identified as female and White and attending 4-year institutions were overrepresented in the sample compared to national enrollment data [47]. The study results must be interpreted with the recognition that not all students, particularly those who may have more vulnerabilities, are equitably represented. Although comparable with other national college health surveys [17,18], the relatively low ACHA-NCHA III response rate could have resulted in a self-selection effect. There is the possibility that students who were more academically driven or who had a particular health status may have been more likely to participate in the survey, influencing the results; however, many schools offered incentives to decrease this kind of bias. Students’ GPA and health behaviors were self-reported, and similar to the original study, social desirability could have affected responses. While self-reported GPA is widely utilized in research and has adequate reliability, using verified university data is ideal as it reduces validity concerns [48,49]. This study was cross-sectional; therefore, directionality between students’ prevention behaviors and GPA is indeterminate and causation can’t be inferred. Although it could be that healthier students perform better academically, it is also possible that high academic performers have more capacity to engage in healthier behaviors. Additionally, because this research modeled the study conducted by Hawkins et al. [14], it examined the same health behaviors related to chronic disease as the original study. Other health behaviors that affect chronic disease risk (e.g., sleep, alcohol consumption) were therefore not included and their relationship with students’ academic achievement in the current study is not known. Finally, the sedentary behaviors reported– specifically watching television and using social media – may not always be sedentary as individuals could watch or scroll while engaging in physical activity, though the same concern exists for the measures used by Hawkins et al. [14].

In spite of these limitations, the results provide guidance for practice. Students’ establishment of health behaviors during college is often predictive of future lifelong health behaviors [2] and GPA is predictive of job performance success [50]. Thus, creating an environment supportive of healthy behaviors and academic achievement is essential for each issue independently and synergistically. For example, one study examining chronic disease within community college students found none of the students diagnosed with cancer or who were prescribed medication for diabetes or a heart problem completed a bachelor's degree within seven years [51]. College health programs must integrate students’ overall well-being – not only behaviors or experiences traditionally thought of as high risk (e.g., alcohol use or sexual assault) – into their efforts to support students. This approach needs to include campus specific assessments and tailored program and policy implementation in collaboration with student success initiatives [1] to best support students’ engagement in chronic disease prevention both now and in the future. The Whole School, Whole Community, Whole Child (WSCC) framework offers institutions of higher education a model for how to accomplish this. WSCC is the U.S. Centers for Disease Control and Prevention’s framework for addressing health in primary and secondary schools. Based on a student-centered ecological approach, the WSCC framework emphasizes the interconnection between students’ health and academic achievement and the need for evidence-based school policies and practices [52,53]. Although institutions of higher education are a distinct setting, the WSCC framework may provide a starting point to further conceptualize the relationship between college students’ health and academic performance and how both can be supported in the absence of an existing framework at the collegiate level.

This study’s results also prompt avenues for additional research. Longitudinal studies that can provide more clarity regarding the potential causative relationship between college students’ health behaviors and academic achievement as well as the actual development of chronic diseases would be beneficial, ideally following students throughout their collegiate experience and afterwards. This approach would also allow for use of other indicators of academic achievement (e.g., graduation, job obtainment) to further understand the interconnection of health, academics, and future success. Additionally, future research should aim to include more representative samples of college students and institutions of higher education, particularly those typically underrepresented (e.g., BIPOC students, community colleges) as well as samples with more GPA variance. Further, as mentioned previously, because this research was based on another study [14], it included the same positive health behaviors and excluded others. Given the growing understanding of additional health behaviors and conditions that also affect chronic disease risk and potentially academic outcomes among college students (e.g., sleep, mental health, alcohol use, food insecurity), a health index specific to the collegiate population may be a worthwhile direction for future investigation.

By engaging in health behaviors that reduce chronic disease risk, college students may also be improving their academic performance and future prospects. The present study indicates that not only are individual positive health behaviors critical – but that the accumulation of healthy behaviors is also interrelated with college students’ academic success. Institutions of higher education should improve alignment among campus stakeholders invested in college students’ health and academic performance to develop or maintain mutually beneficial programs and policies. While more research is needed to understand causal connections, the current study provides a post-COVID-19 understanding about chronic disease prevention behaviors and academic success as a foundation for that future work.
